# Iron Accumulation Is Not Homogenous among Patients with Parkinson's Disease

**DOI:** 10.1155/2015/324843

**Published:** 2015-04-05

**Authors:** Khashayar Dashtipour, Manju Liu, Camellia Kani, Pejman Dalaie, Andre Obenaus, Daniel Simmons, Nicole M. Gatto, Mehran Zarifi

**Affiliations:** ^1^Department of Neurology, School of Medicine, Loma Linda University, Loma Linda, CA 92354, USA; ^2^Department of Radiology, School of Medicine, Wayne State University, Detroit, MI 48202, USA; ^3^Department of Pediatrics, School of Medicine, Loma Linda University, Loma Linda, CA 92354, USA; ^4^Center for Nutrition, Healthy Lifestyles and Disease Prevention, School of Public Health, Loma Linda University, Loma Linda, CA 92354, USA

## Abstract

*Background*. Iron is considered to lead to neurodegeneration and has been hypothesized as a possible cause of Parkinson's disease (PD). Susceptibility-weighted imaging (SWI) is a powerful tool to measure phase related iron content of brain. *Methods*. Twelve de novo patients with PD were recruited from the Movement Disorders Clinic, Department of Neurology, Loma Linda University. Twelve age- and sex-matched non-PD subjects were recruited from neurology clinic as controls. Using SWI, the phase related iron content was estimated from different brain regions of interest (ROIs). *Results*. There was a trend between increasing age and iron accumulation in the globus pallidus and putamen in all subjects. Iron accumulation was not significant in different ROIs in PD patients compared to controls after adjustment for age. Our data revealed heterogeneity of phase values in different brain ROIs among all subjects with an exaggerated trend at SN in PD patients. *Conclusions*. Our data suggest a nonhomogeneous pattern of iron accumulation in different brain regions among PD patients. Further studies are needed to explore whether this may correlate to the progression of PD. To our knowledge, this is the first study demonstrating the heterogeneity of iron accumulation in the brain, among patients with PD.

## 1. Introduction

The correlation between iron and neurodegenerative disorders including Parkinson's disease (PD) dates back to 1924, when Lhermitte et al. demonstrated accumulation of iron in brain autopsies [1]. Since then, multiple studies have replicated those results [2–6] and raised the question regarding the primary versus secondary role of iron in neurodegenerative disorders. Specifically, it remains to be established whether iron triggers the neurodegenerative process or whether the accumulation of the iron is secondary to the neuronal damage and cell loss. Currently, it is accepted that iron is cytotoxic and is able to generate oxygen free radicals [7]. A direct role of iron in neurodegeneration has been supported by animal studies that demonstrate where iron chelators protect rats against 6-hydroxydopamine (6-OHDA) induced striatal lesions [8]. Interestingly, direct injection of iron into the substantia nigra (SN) elicits parkinsonian like features in rats [9]. Conversely, iron does not readily cross the blood brain barrier, and as such, accumulation of iron in certain brain regions in PD might suggest its translocation from one anatomical location to another region of the brain [10].

Furthermore, the interpretation of observed increases of iron in the brains of patients with PD has been questioned after some studies could not replicate the results from previous research [11, 12]. Subsequently, however, reviews of these studies' methodology revealed that the lack of concordant results was most likely due to dissimilarities in tissue preparation and fixation techniques [11].

Multiple imaging techniques have been used to establish correlations between the magnetic resonance (MR) signal and iron content. Increased T2, T2^*^, and Tr′ field dependent relaxation rates and susceptibility weighted imaging (SWI) have shown to be powerful tools to measure iron content of the human brain [13–18], and their resulting measures have been interpreted as surrogate indices of brain iron. Recently, several studies have attempted to assess brain iron levels in vivo in patients with PD [13–25]. Most but not all studies suggest higher iron content in the SN of patients with PD compared to unaffected control subjects. However, these results have not been replicated between studies, and no single study was able to identify a threshold value of iron that could be used as a biomarker to distinguish between patients with PD and controls.

In the current study, we examined whether newly diagnosed patients with PD exhibit higher brain iron content in comparison to non-PD subjects measured by SWI.

## 2. Material and Methods

### 2.1. Subjects

Twelve patients with a new diagnosis of PD were recruited by movement disorders specialists at Loma Linda University Medical Center (LLUMC) neurology clinic. PD diagnosis was established based on UK PD Society Brain Bank Criteria [26]. Atypical parkinsonism and patients with concomitant vascular parkinsonism or parkinsonism secondary to neuroleptics were initially ruled out in all twelve subjects. Twelve non-PD subjects who were examined by a neurologist at LLUMC neurology clinic and were verified not to have PD were also recruited. The non-PD subjects suffered from various neurological disorders such as tardive syndrome, migraine headache, essential tremor, enhanced physiological tremor, neuropathy, myoclonus, and focal dystonia. None of the PD or control subjects had a current or prior history of cognitive impairment, stroke, brain lesions, or other neurodegenerative disorders.

### 2.2. MR Imaging Protocol

All of the twenty-four subjects underwent magnetic resonance imaging (MRI) on a 3T Siemens magnet using a 12-channel head coil (12 head matrix). PD subjects were imaged 1–3 months after diagnosis of PD was established. The scan parameters were as follows: TE = 20 ms, TR = 29 ms, FA = 15, matrix = 448 × 336, resolution  =  0.5  ×  0.5  ×  2 mm^3^, pixel bandwidth = 100, and FOV = 230 × 187 mm. Phase images were filtered using a 96 × 96 high pass filter, which is embedded in the Siemens reconstruction software.

### 2.3. Regions of Interest (ROIs)

**Figure 1:**
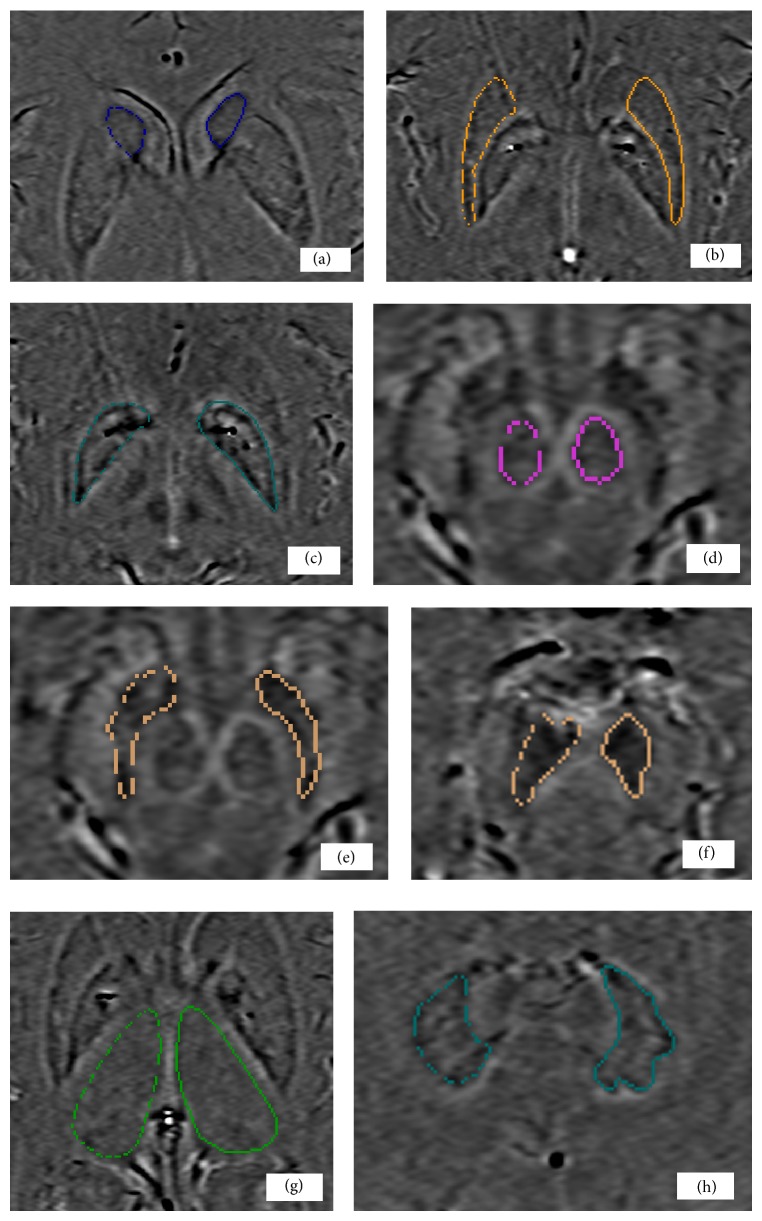
Regions of interest drawn on SWI: CN (a), PUT (b), GP (c), RN (d), SN (e), caudal SN (f), THA (g), and DN (h). CN: caudate nucleus, PUT: putamen, GP: globus pallidus, RN: red nucleus, SN: substantia nigra, caudal SN: caudal substantia nigra, THA: thalamus, and DN: dentate nucleus.

Two independent investigators blinded to patient or control status drew the ROIs (Figure 1). The SN was identified on the third slice caudally, after the appearance of the red nucleus. For the SN, two areas were drawn: the entire SN overall and the caudal SN. Gray matter (GM) was identified as motor cortex and the white matter (WM) was identified as areas adjacent to the gray matter. For red nucleus (RN), putamen (PUT), globus pallidus (GP), caudate nucleus (CN), dentate nucleus (DN), and thalamus (THA), the optimum slice was selected with reference to standard neuroanatomic criteria [27].

### 2.4. Data Analysis

Data processing was done using Signal Processing in Nuclear Magnetic Resonance software (SPIN, MRI Institute, Detroit, Michigan). Average phase values within each ROI were calculated. To eliminate the effect of iron content difference due to the overall iron level difference for a given subject, we normalized each phase measurement to the overall brain iron content level, which was represented by averaging the phase measurements for all the ROIs of each subject (average-scale = [average phase/overall iron level] × 1000).

### 2.5. Statistical Analysis

Nonparametric *t*-tests were used to determine significant difference between the two groups. Given the exploratory nature of this analysis, a *P* value of 0.05 for the difference between phase values in PD patients and controls was accepted as statistically significant. Spearman correlation analysis was conducted to analyze the relationship between the age and phase values. Age was modeled as a covariate to correct for age-related iron deposition by the binary logistic regression analysis. To assess agreement between both observers, Bland-Altman plot was constructed. All statistics analyses were performed using SPSS statistical software (version 13.0, SPSS Inc., Chicago, Illinois).

## 3. Results

### 3.1. Demographic and Clinical Characteristics

Subjects' demographic and clinical characteristics have been shown in Table 1.

### 3.2. Iron Content in Various ROIs

Table 2 summarizes iron values for the ten ROIs by patient or control status. The average iron levels were not significant in different ROIs in PD patients compared to control subjects.

The SN and caudal aspect of the SN showed nearly significant increased iron deposition in the patient group compared to controls. After adjustment for age, however, none of these regions remained significant.

In both groups, we found a significant positive correlation between age and the average iron level in the GP (*r* = 0.496, *P* = 0.016) and marginally significant correlation between age and the average iron level in the PUT (*r* = 0.396, *P* = 0.051). Age in PD patients was found to be positively correlated with the average iron deposition in the THA (*r* = 0.723, *P* = 0.008). There was also a trend between increasing age and iron deposition in the SN (*r* = 0.540, *P* = 0.060) in PD patients.

### 3.3. Phase Analysis among Subjects

**Figure 2:**
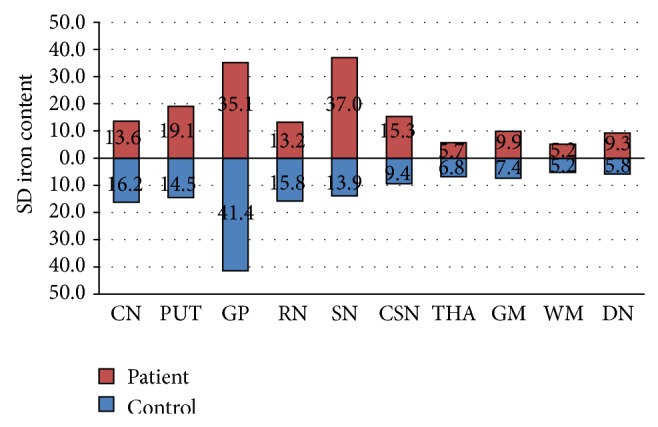
Heterogeneity of phase values at different ROIs with an exaggerated trend at SN in PD patients.

**Figure 3:**
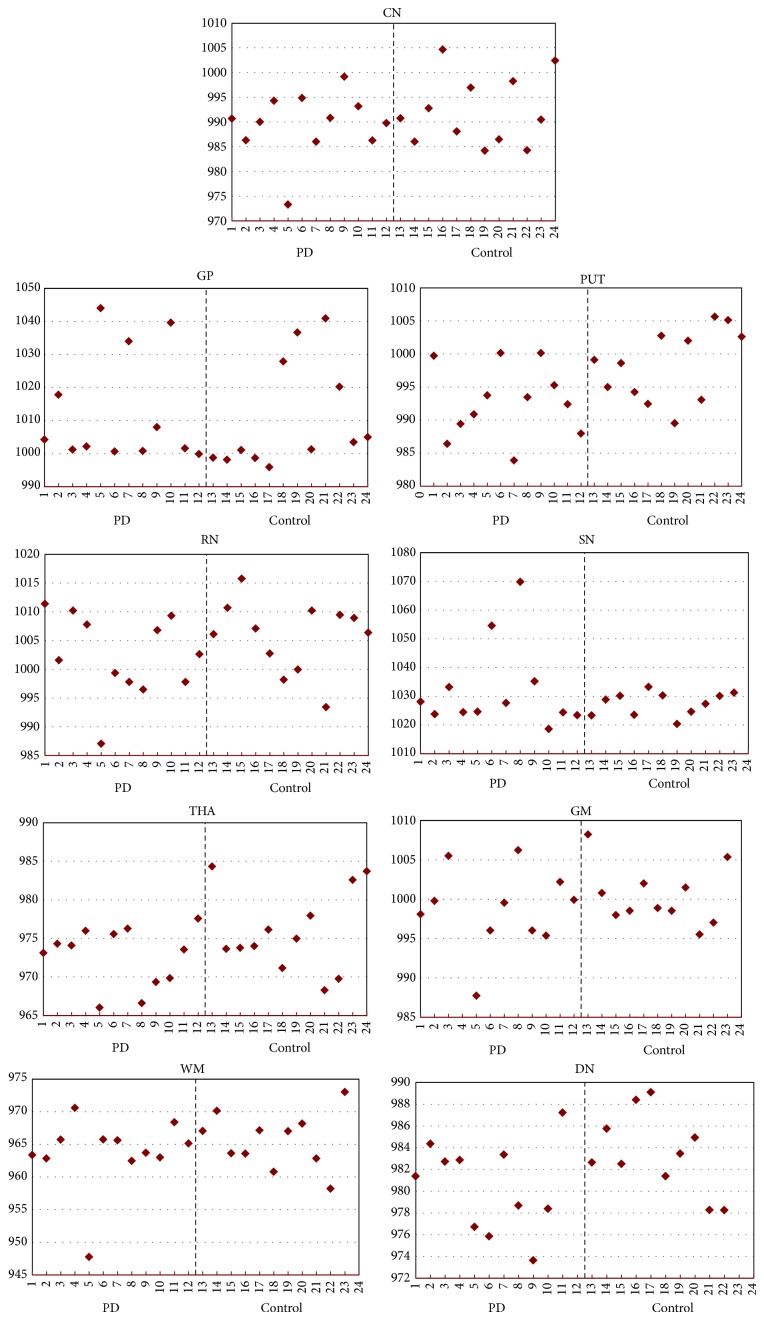
Heterogeneity of phase values in various brain regions among study subjects. CN: caudate nucleus, PUT: putamen, GP: globus pallidus, RN: red nucleus, SN: substantia nigra, THA: thalamus, GM: gray matter, DN: dentate nucleus, and WM: white matter.

To assess the heterogeneity of iron accumulation in different ROIs, the standard deviation (SD) of phase values at each region was calculated (Table 2). The results revealed heterogeneity of phase values for iron at different ROIs in all subjects with an exaggerated trend at SN in PD patients (Figures 2 and 3).

Not one of the twelve patients had the same pattern of high iron content in the same brain region (Figure 3).

We did not find any laterality on iron deposition and distribution despite unilateral symptoms at the onset in the patients (Table 3).

### 3.4. Interrater Reliability

Pearson's correlation coefficients for the phase values of the selected ROIs showed good agreement between blinded raters with differences of less than 0.05 radians.

## 4. Discussion

The key result of this study indicates that accumulation of iron in different brain regions is not homogenous among patients with PD and that certain phase thresholds within these brain regions may be useful as to differentiate PD from controls.

### 4.1. Role of the Iron in Neurodegeneration

Although older studies have questioned the role of iron in neurodegenerative disorders such as PD the advent of improved imaging techniques in vivo and of autopsy tissue preparations argues in favor of a role for iron in neurodegeneration [2–5]. Based on the role of iron in the Fenton reaction and generating detrimental free radicals, the role of iron in PD has already been discussed in several review articles [3, 7, 10]. Brain iron metabolism results in oxygen free-radical-induced oxidative stress. Two oxygen free-radical species have been identified to damage biological systems which are superoxide and hydroxyl free radical. Fe^3+^^.^ Fe^2+^ plays an essential role in the formation of hydroxyl free radical from H_2_O_2_ and superoxide. Consequences of free radical generation include a series of reactions ending in lipid peroxidation of cell membranes, followed by membrane fluidity, and ultimately in cell death [7, 10]. In PD, the melanized nigrostriatal dopamine neurons of SN, where iron is increased, degenerate selectively. Here we showed a trend for increasing iron accumulation in SN among PD patients compared to control subjects.

### 4.2. Brain Iron as Biomarker in PD

A reliable biological marker to help clinicians confirm the diagnosis of PD and measure the progression of the disease is currently an unmet need. The diagnosis of PD is based on neurologically observable clinical signs and symptoms, with a definitive diagnosis provided solely at autopsy. Ideally, a reliable biomarker should be able to identify individuals at risk before the onset of motor symptoms in PD and accurately diagnose individuals at the threshold of clinical PD. Such a biomarker should also be able to monitor PD progression throughout its course and objectively measure and evaluate responses to therapeutic interventions. To have the above features, the potential biomarker should be related to the mechanisms of the disease and its pathophysiology. Pathological studies from autopsies of patients with PD have provided evidence of increased brain iron accumulation in PD and raised the possibility of its use as a biomarker [2–7]. Whether or not the role of iron is primary in the pathophysiology of the disease or its accumulation is secondary to the cell loss is still debatable. But it seems there is strong evidence to support its positive correlation with the disease state. For this reason, recent multiple neuroimaging techniques have been applied to measure brain iron levels in vivo [13–25]. Similar to previous studies, we utilized SWI and used quantitative phase (radians) as a surrogate measure of brain iron levels. We have replicated results of the previous studies by showing a trend for higher iron concentration in SN compared to the control group.

### 4.3. Heterogeneity of the Iron Accumulation among PD Patients

An interesting and innovative observation in our study was the observation of heterogeneity of brain iron accumulation among PD subjects (Table 3 and Figure 2). Despite the heterogeneity of iron content from imaging, all twelve PD patients exhibited very similar clinical signs at the time of diagnosis with unilateral symptoms and all presenting with bradykinesia, rigidity, and resting tremor. It is well known that patients with PD can progress differently with a multitude of clinical complications that can result in dyskinesia, motor fluctuation, gait impairment, and nonmotor complications including cognitive decline and hallucination, to name a few. We found the heterogeneity of iron accumulation in the brain of PD patients very interesting since the pattern of iron accumulation might be used in predicting the clinical outcome of the disease. Recently Rossi et al. in a 2-year followup study of 25 patients with PD detected that the rate of iron changes was associated with individual characteristics such as cognitive decline and age at disease onset [23]. Clearly, further larger longitudinal studies are needed to test our hypothesis regarding the heterogeneity of iron accumulation and progression of the disease. To our knowledge, this study is the first study demonstrating this heterogeneity among PD patients.

*Limitations of the Study*. The small sample size in relation to the number of statistical tests conducted is a limitation of this study. Nevertheless, the analysis was exploratory and results should be confirmed by additional studies. The average age of PD patients is almost 10 years older than the control subjects and age plays a significant role in brain accumulation of iron. All cases were diagnosed by at least one movement disorder specialist based on UK PD Society Brain Bank Criteria; however, there was no definitive pathological evidence to confirm our PD diagnosis. Normal brain iron varies by race/ethnicity and can be associated with environmental factors such as diet. All patients included in the current study were de novo cases and they had not received dopaminergic therapy before the first SWI MRI. As phase can be influenced by dopaminergic therapy and disease progression, additional followup studies are needed to further elucidate these influences.

## 5. Conclusion

Our data suggest that PD patients manifest a nonhomogeneous pattern of iron accumulation in brain, and this pattern is different among PD patients. Further studies are needed to explore whether these findings correlate with the progression of PD. To our knowledge, our study is the first to demonstrate the heterogeneity of iron accumulation among PD patients.

## Figures and Tables

**Table 1:** Demographic and clinical characteristics of subjects.

	PD	Control
	*N* = 12	*N* = 12
Male : female	3 : 9	4 : 8
Mean age ± SD (years)^*^	72.67 ± 9.01	63.33 ± 5.97
Hoehn and Yahr Stage	1.5	NA
Time of diagnosis to MRI (months)	2.2 ± 0.3	NA

NA: not applicable, SD: standard deviation.

^*^ *P* < 0.05.

**Table 2:** Average phase value for each brain region after adjustment for age.

Average phase value (average of left and right sides)	Patients *N* = 12	Controls *N* = 12	*P* value^*^
	Mean (SD)	Mean (SD)	
CN	2159.96 (13.59)	2159.07 (16.16)	0.885
PUT	2167.04 (19.06)	2172.58 (14.47)	0.432
GP	2202.03 (35.09)	2199.53 (41.45)	0.878
RN	2187.85 (13.18)	2188.70 (15.81)	0.887
SN	2253.34 (36.97)	2238.41 (13.91)	0.222
C-SN	2233.12 (15.32)	2226.35 (9.41)	0.239
THA	2123.16 (5.69)	2123.67 (6.84)	0.844
GM	2181.32 (9.91)	2179.21 (7.35)	0.576
WM	2103.51 (5.15)	2103.44 (5.15)	0.975
DN	2141.10 (9.34)	2144.15 (5.85)	0.387

^*^Mann-Whitney *U* test.

CN: caudate nucleus, PUT: putamen, GP: globus pallidus, RN: red nucleus, SN: substantia nigra, C-SN: caudal substantia nigra, THA: thalamus, GM: gray matter, WM: white matter, and DN: dentate nucleus.

**Table 3:** Different ROIs with high phase values among PD subjects.

PD patients	ROI with highest iron	Age (year)	Sex (M/F)	Phase value Average of ROIs		Phase value SN		Presenting symptoms		
				R	L	R	L	Bradykinesia	Tremor	Rigidity
1	RN	70	F	2160.31	2167.95	2221.50	2253.20	L	L	L > R
2	DN	69	F	2165.37	2169.61	2236.43	2232.80	R > L	R, Chin	R > L
3	GM, RN	54	F	2164.15	2161.73	2254.30	2239.10	R	R, Chin	R
4	WM	65	F	2162.35	2166.62	2214.00	2229.80	R	—	R < L
5	GP	87	F	2203.23	2215.00	2273.56	2258.32	R~L	R	—
6	SN, PUT	82	F	2173.01	2170.20	2309.36	2289.93	L	L	L
7	GP	76	F	2161.37	2170.46	2235.00	2239.55	R	R	R
8	GM, SN	79	M	2179.54	2190.01	2307.74	2384.97	R	R	R
9	PUT, CN	76	F	2176.81	2179.75	2253.78	2280.21	L	L	L
10	GP	75	M	2187.80	2177.65	2251.21	2214.00	L > R	R	L
11	WM, DN	63	M	2159.29	2158.28	2238.00	2217.60	R	R	R
12	THA	76	F	2165.38	2167.63	2214.00	2232.00	L	L	L

CN: caudate nucleus, GP: globus pallidus, PUT: putamen, RN: red nucleus, SN: substantia nigra, THA: thalamus, C-SN: caudal substantia nigra, DN: dentate nucleus, WM: white matter, GM: gray matter, M: male, F: female, L: left, and R: right.
